# Insular Cortex Modulation by Repetitive Transcranial Magnetic Stimulation with Concurrent Functional Magnetic Resonance Imaging: Preliminary Findings

**DOI:** 10.3390/brainsci15070680

**Published:** 2025-06-25

**Authors:** Daphné Citherlet, Olivier Boucher, Manon Robert, Catherine Provost, Arielle Alcindor, Ke Peng, Louis De Beaumont, Dang Khoa Nguyen

**Affiliations:** 1 Neurosciences Axis, Centre de Recherche du Centre Hospitalier de l’Université de Montréal (CRCHUM), Montreal, QC H2X 0A9, Canada; daphne.citherlet@umontreal.ca (D.C.); olivier.boucher.chum@ssss.gouv.qc.ca (O.B.); manon.robert.chum@ssss.gouv.qc.ca (M.R.); arielle.alcindor@umontreal.ca (A.A.); 2 Department of Neurosciences, University of Montreal, Montreal, QC H2V 0B3, Canada; 3 Department of Psychology, University of Montreal, Montreal, QC H2V 0B3, Canada; 4 Centre de Recherche de l’Hôpital du Sacré-Cœur de Montréal, Montreal, QC H4J 1C5, Canada; catherineprovost@hotmail.ca (C.P.); louis.de.beaumont@umontreal.ca (L.D.B.); 5 Department of Electrical and Computer Engineering, University of Manitoba, Winnipeg, MB R3T 5V6, Canada; pengke04@gmail.com; 6 Department of Surgery, University of Montreal, Montreal, QC H3C 3J7, Canada; 7 Neurology Division, Centre Hospitalier de l’Université de Montréal (CHUM), Montreal, QC H2X 0C1, Canada

**Keywords:** insular cortex, rTMS, fMRI, neuromodulation, neurostimulation, high-frequency, dysgeusia, BOLD signal, neuronavigation, concurrent rTMS-fMRI

## Abstract

Background/Objectives: The insula is a deep, functionally heterogeneous region involved in various pathological conditions. Repetitive transcranial magnetic stimulation (rTMS) has emerged as a promising therapeutic avenue for neuromodulation, yet very few studies have directly investigated its effects on insular activity. Moreover, empirical evidence of target engagement of this region remains scarce. This study aimed to stimulate the insula with rTMS and assess blood oxygen level-dependent (BOLD) signal modulation using concurrent functional magnetic resonance imaging (fMRI). Methods: Ten participants were recruited, six of whom underwent a single session of 5 Hz high-frequency rTMS over the right insular cortex inside the MRI scanner. Stimulation was delivered using a compatible MRI-B91 TMS coil. Stimulation consisted of 10 trains of 10 s each, with a 50 s interval between trains. Frameless stereotactic neuronavigation ensured precise targeting. Paired t-tests were used to compare the mean BOLD signal obtained between stimulation trains with resting-state fMRI acquired before the rTMS stimulation session. A significant cluster threshold of q < 0.01 (False Discovery Rate; FDR) with a minimum cluster size of 10 voxels was applied. Results: Concurrent rTMS-fMRI revealed the significant modulation of BOLD activity within insular subregions. Increased activity was observed in the anterior, middle, and middle-inferior insula, while decreased activity was identified in the ventral anterior and posterior insula. Additionally, two participants reported transient dysgeusia following stimulation, which provides further evidence of insular modulation. Conclusions: These findings provide preliminary evidence that rTMS can modulate distinct subregions of the insular cortex. The combination of region-specific BOLD responses and stimulation-induced dysgeusia supports the feasibility of using rTMS to modulate insular activity.

## 1. Introduction

The insula is a deep structure located behind the frontal, parietal, and temporal opercula. Anatomically, it is divided into two portions by the central insular sulcus: the anterior insula (aI) and the posterior insula (pI) [1,2]. Functionally, the insula is divided into three subregions: the mid-posterior insula, and the ventral and dorsal aI. The insula has been implicated in a variety of functions, including autonomic and vestibular functions, interoception, sensory, cognitive, and affective processes [3,4,5,6,7,8]. The mid-posterior insula is connected to primary and secondary somatosensory cortices, whereas the dorsal aI is connected to the dorsal anterior cingulate cortex (dACC) and prefrontal cortex (PFC). The ventral aI shows functional connectivity with the inferior frontal gyrus and the temporal lobe including the amygdala [9]. Furthermore, the insular cortex plays a central role in several pathologies, such as obesity [10,11,12,13,14] and chronic pain [15,16,17,18]. These conditions are often characterized by limited responses to conventional treatments. In this context, neuromodulation has emerged as a promising therapeutic strategy for patients who do not respond adequately to traditional pharmacological or psychotherapeutic interventions [19].

Repetitive transcranial magnetic stimulation (rTMS) is a non-invasive neurostimulation technique that appears to be promising for the treatment of several medical conditions. Technically, rTMS modulates neuronal activity by inducing electrical currents through time-shifting magnetic field pulses [20]. High-frequency rTMS has been widely used in the treatment of depression, mainly targeting the left dorsolateral PFC (DLPFC) [21,22,23,24]. It has also been shown to be safe and potentially effective in the modulation of craving, alcohol and cigarette consumption, and decision-making in addiction when applied to the DLPFC, medial PFC, and dACC [25,26,27,28,29,30,31,32,33,34,35,36]. A recent review reported significant analgesic effects of motor cortex rTMS in neuropathic pain, supported by 14 randomized placebo-controlled trials involving approximately 750 patients [37]. The pI is a promising target, but current evidence remains limited. Finally, a systematic review found that high-frequency rTMS targeting the bilateral DLPFC and the insula was associated with the greatest reduction in body mass index [38].

To date, only a limited number of studies have examined the insula as a therapeutic target. The promising results are not unexpected, however, given the central role of this structure in several clinical conditions. Spagnolo and colleagues [39] reported that low-frequency deep rTMS at an intensity of 120% of the individual’s motor threshold (MT) over the right aI, using an H8 coil, was safe but did not affect performance on behavioural tasks, highlighting that high-frequency stimulation may be required to effectively activate the insula. In 2017, a clinical trial explored the potential of targeting the insula in the treatment of addiction. The study aimed to measure changes in dopamine levels using Positron Emission Tomography (PET) with [11C]-(+)-propyl-hexahydro-naphtho-oxazin. Participants underwent three PET scans after different rTMS sessions (sham, 1 Hz, or 10 Hz). The results showed that low-frequency rTMS targeting the insula significantly decreased dopamine levels in the substantia nigra, sensorimotor striatum, and associative striatum [40]. Dinur-Klein and colleagues [41] showed that high-frequency deep rTMS of the PFC and insula bilaterally reduced cigarette consumption and nicotine dependence, in contrast to low-frequency or sham treatments, and appears to be a promising treatment strategy for addiction. These findings are consistent with Ibrahim and colleagues, who reported the usefulness of combining deep insula rTMS with medication to improve smoking abstinence rates [42]. In addition, the deep continuous theta-burst stimulation of the right operculo-insular cortex selectively impaired the perception of thermonociceptive input from Aδ-fibre thermonociceptors, without affecting the perception of innocuous warm, cold, or vibrotactile sensations [43]. Another study reported subjective changes in cold perception following rTMS over the posterior-superior insula at a frequency of 10 Hz with an intensity at 80% of MT [44]. In addition, deep posterior-superior insula rTMS was associated with a significant reduction in pain intensity in refractory peripheral neuropathic pain [45]. Finally, a pilot study suggested that deep rTMS over the insula was safe, effective, and well tolerated in patients with anorexia nervosa [46]. While these studies targeting the insula have shown promising results, a major challenge remains in determining whether stimulation effectively reaches the insular cortex due to its deep location within the Sylvian fissure. This anatomical depth presents a significant obstacle for conventional non-invasive neuromodulation techniques, which usually target more superficial cortical regions. Additionally, our understanding of the neuromodulatory impact of the insula is limited by the lack of objective measures to verify stimulation effects. Therefore, the accurate placement of the TMS coil using a neuronavigation system, which incorporates anatomical MRI and the real-time tracking of the coil’s position, is therefore crucial to ensure the precise targeting of cortical regions.

To address these challenges, the present study combines simultaneous rTMS and functional magnetic resonance imaging (fMRI) to enable the direct, real-time observation of the insula’s response to stimulation. Integrating neuromodulation with neuroimaging enhances spatial precision and provides new insights into the functional modulation within the insula and neighbouring regions, thereby filling an important methodological gap. Additionally, frameless stereotactic neuronavigation using individual MRI data is essential for precisely positioning the TMS coil on the scalp.

Our study aimed to assess the feasibility of modulating insular activity using high-frequency rTMS and to determine the optimal rTMS parameters required to effectively modulate insular activity. rTMS was applied directly within the MRI scanner, providing a direct measure of neuronal modulation. This simultaneous rTMS-fMRI integration allowed the characterization of changes in local cerebral blood flow and oxygenation, which in turn modulated the blood-oxygenation-level-dependent (BOLD) imaging signal [47].

## 2. Materials and Methods

Participants: Ten healthy participants (five women and five men), all right-handed, were recruited in this study. Inclusion criteria required that participants had no significant health problems, no diagnosis of neurological and/or psychiatric disorders, and no condition that would make MRI and rTMS unsafe (e.g., metallic implants or pacemakers). Furthermore, none of the participants were undergoing any concurrent treatments, including pharmacological, psychological, or neuromodulatory interventions, at the time of the study to avoid potential confounding effects on the BOLD signal. Prior to rTMS modulation, each participant underwent an individual T1-weighted MRI scan on a separate day to rule out any medical conditions that might affect participation. One participant was excluded due to structural brain abnormalities (SBJ8). Another participant initially agreed to participate in the rTMS but later declined (SBJ7). A technical issue with the coil prevented us from performing stimulation in one participant (SBJ10). We had to interrupt the stimulation in another participant because of severe pain in the right temple caused by the pulse administration from the TMS coil (SBJ5). Thus, a total of six participants (four women and two men) received rTMS modulation over the right insular cortex with simultaneous fMRI recording. This study was approved by the CHUM ethics committee (2019-7917, 18.122). All participants gave informed consent prior to all study procedures.

**Figure 1:**
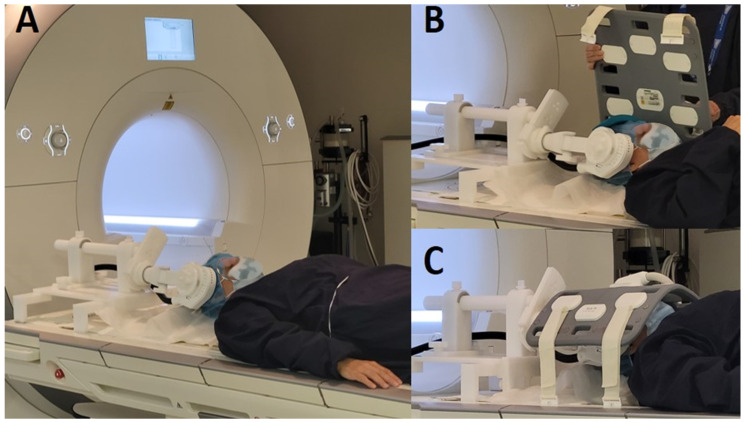
The MRI-B91 coil is attached to an MRI-compatible TMS support while the subject lies on the MRI table, with the coil positioned on the scalp (**A**). A 16-channel head antenna is placed over the participant’s head to optimize signal reception during the fMRI acquisition (**B**,**C**).

Identification of the target: To target the insula using frameless stereotactic neuronavigation, the previously acquired anatomical MRI of each participant was integrated into the Brainsight system, which generates a 3D model of the brain (Brainsight system^®^, Polaris system, Rogue Research, Montréal, QC, Canada). Four parts of the insula were identified, namely, the limen insula, the superior border/temporal operculum, the anterior border/frontal operculum, and the posterior border/parietal, based on anatomical landmarks described by Ciampi de Andrade and colleagues [44]). Real-time tracking was used to monitor the position of the TMS coil over the participant’s head while they lay on the MRI table. At this stage, the MRI table was not yet positioned inside the scanner. The coil was attached to the TMS support, which is compatible with fMRI and enables the stable positioning of the coil during simultaneous stimulation and fMRI (see Figure 1). We used three tracking tools: the subject tracker, positioned on the participant’s forehead; the coil tracker, attached directly to the TMS coil to guide the coil handle relative to the magnetic field hotspot; and the pointer tool, for precise anatomical localization. Neuronavigation enabled millimetric precision in aligning the coil with the intended insular targets during the setup phase. The TMS coil was positioned on the right side for all participants. To ensure consistent coil placement across participants and minimize variability related to functional asymmetries between the hemispheres, we selected the right side. Targeting the right hemisphere reduces potential confounds associated with language processing, which is predominantly lateralized to the left hemisphere in most individuals. Due to the difficulty of stimulating a deep cortical structure like the insula, we optimized the coil’s angulation within the physical constraints of the MRI environment and customized the coil placement based on each participant’s unique anatomy. Particular attention was given to targeting the more accessible operculo-insular subregions of each participant. Head movement was restricted by foam-padded cushions. Once the coil was correctly positioned over the insula, the subject was moved into the MRI scanner with the TMS coil attached to the MRI-compatible support. Table 1 and Table 2 show each participant’s individual motor threshold (MT) and the targeted insular subregions.

rTMS procedure: We used the MRI-B91 coil with compressed air cooling (Magventure^®^, MagProX100, Farum, Denmark). This coil generates a magnetic field of 3 to 6 kT/s for deep stimulation (3–5 cm) at 100% stimulator intensity and is compatible with MRI. Individual MT was determined by stimulating the left primary motor cortex and identifying the minimum intensity required to activate the right abductor pollicis brevis muscle in at least 6 out of 10 trials. High-frequency stimulation was performed with 10 trains of 10 s each at 5 Hz, with a 50 s interval between trains. Each stimulation train consisted of 50 pulses, for a total of 500 pulses. Stimulation intensity was delivered between 60% and 75% of the individual MT, depending on the participant’s tolerance for discomfort during repetitive pulses. Several considerations guided the choice of stimulation parameters. First, our protocol was based on the study by Ciampi de Andrade et al. [44] which successfully used high-frequency rTMS to modulate operculum-insular activity. This supports the use of excitatory stimulation for the target region. Second, we adapted the protocol to accommodate the technical constraints of the MRI environment, including coil heating limitations and positioning restrictions. Third, we adhered to international safety recommendations for rTMS [48,49,50] to ensure that the stimulation frequency, train duration, and total number of pulses remained within the established safety thresholds. Finally, we adjusted the stimulation intensity individually (60–75% MT) to account for each participant’s tolerance and comfort. This is particularly important in MRI-compatible setups using cooled coils.

fMRI image acquisition: All participants underwent fMRI acquisition simultaneously with rTMS. Prior to this, a resting-state functional MRI (RS-fMRI) was performed without rTMS. A 3 Tesla whole-body scanner (Skyra, Siemens) with a 16-channel head antenna was used to acquire fMRI data. We used a T2*-weighted gradient echo planar imaging sequence (i.e., slices = 49; TR = 3000 ms; TE = 30 ms; flip angle = 90°; FOV phase = 100%; FOV read = 230 mm; matrix = 64 × 64 matrix; voxel resolution = 2.4 × 2.4 × 3 mm; slice thickness = 3 mm). Participants were instructed to keep their eyes open during the rTMS-fMRI acquisition and to wear earplugs throughout the experiment.

fMRI data processing: The alignment between fMRI slices and rTMS was ensured by synchronizing the onset of rTMS with the fMRI acquisition. We precisely calculated the timing of each stimulation event relative to the fMRI recording. Several parameters were used to determine the onset of rTMS relative to the MRI scans, including the total number of slices, the repetition time (i.e., TR), the echo time (i.e., TE), and the duration of the stimulation trains. This approach allowed us to align rTMS events with specific fMRI slices. We took great care to minimize artefacts from the simultaneous use of TMS and MRI. However, our fMRI data were noisy due to artefacts when TMS pulses occurred. Therefore, we decided to restrict analysis to MRI slices obtained in between stimulations and exclude the MRI slices acquired during stimulations from further analysis. We used Statistical Parametric Mapping software (SPM12, r7771) on MATLAB (MATLAB software R2022b) for fMRI data processing. Motion correction was performed using SPM’s Realign function, which registered all functional images to a reference image (i.e., the average of all images). The corrected images were visually inspected to confirm the effectiveness of the motion correction. Slice timing correction was then performed. The anatomical MRI was then normalized to the Montreal Neurological Institute (MNI) brain template. Coregistration was performed to align the functional images with the high-resolution anatomical MRI images of each individual subject. Finally, the coregistered functional images were normalized to the MNI standard brain template.

Statistical analysis: Statistical analyses were performed using SPM12 software and xjView toolbox (https://www.alivelearn.net/xjview/ (accessed on 21 December 2023)). Analyses focused on the fMRI images obtained between rTMS trains. Specifically, paired t-tests were performed to compare the BOLD signal between the average of all fMRI images obtained between stimulation trains and the RS-fMRI images (i.e., RS-fMRI images acquired without the rTMS procedure) using a *p*-value of 0.01. The interval between stimulation trains was defined as the period from 20 to 50 s after the onset of the rTMS trains. This was considered the optimal choice based on increased statistical power. We then used the xjView toolbox to show insular activation and deactivation patterns. The initial voxel-wise threshold was set at *p* < 0.001 uncorrected. Significant clusters were then identified using the False Discovery Rate (FDR) correction at q < 0.01, with a minimum cluster size threshold of 10 voxels. Due to the insufficient number of fMRI images per rTMS train, it was not possible to make comparisons between different stimulation trains or to visualize the time course of BOLD changes after rTMS. For better understanding, the results are presented on the MNI brain template.

## 3. Results

**Figure 2:**
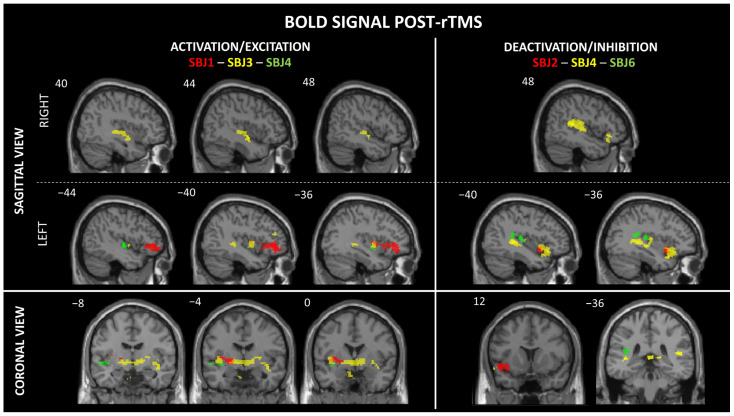
Change in BOLD activity during post-train intervals compared to the resting-state fMRI. Results are displayed at q < 0.01 or q < 0.001, FDR-corrected. The left side represents BOLD signal activation/excitation (SBJ1, SBJ3, and SBJ4) and the right side represents BOLD signal deactivation/inhibition (SBJ2, SBJ4, and SBJ6). From top to bottom, a sagittal view (**right** and **left**), followed by a coronal view. The coordinates are reported in MNI space. In the sagittal view, x = 0 corresponds to the midline. In the coronal view, y = 0 is the anterior commissure.

One subject’s data was excluded from analysis due to significant noise contamination that compromised data quality (SBJ9). For the remaining five participants, we identified between one and two significant activation clusters per subject that survived FDR correction. These activations were characterized by both increases and decreases in BOLD signal, indicating neural responses to stimulation. Specifically, we observed activation in the left (L) middle/aI (SBJ1), L middle insula (SBJ1 and SBJ3), L mid-inferior insula (SBJ4), L pI (SBJ6), and right (R) mid-inferior insula (SBJ3). Deactivation was observed in the L ventral aI/aI (SBJ2 and SBJ4) and the R and L pI (SBJ4) (see Table 2 and Figure 2). In addition, two participants experienced a metallic taste immediately after rTMS. Significant activations were also observed in other brain regions, including the frontal (L and R inferior frontal gyrus and L middle frontal gyrus), temporal (L superior temporal gyrus and L temporal pole), parietal (R supramarginal gyrus and R post-central gyrus), R posterior cingulate, L and R putamen, L parahippocampal gyrus, and L and R globus pallidus regions (see Appendix A, Table A1).

Due to the variability in stimulation targets across participants, group analyses were not performed. Heterogeneity in target locations would have introduced significant variability, complicating group-level inferences and potentially masking meaningful patterns of activation.

## 4. Discussion

In this study, we investigated the feasibility of modulating insular activity using rTMS with concurrent fMRI recording. A single high-frequency 5 Hz rTMS session was administered to six healthy participants over the R insula. Concurrent fMRI revealed significant BOLD signal modulations characterized by increased activity (i.e., activation and positive BOLD signal) in the L middle insula, L and R mid-inferior insula, and L pI subregions. Significant decreased activity (i.e., deactivation and negative BOLD signal) was observed in the L ventral aI, and L and R pI. Two participants reported dysgeusia (i.e., metallic taste). High-frequency stimulation with the MRI-B91 coil over the insular subregions was generally well tolerated despite the limited sample size of this preliminary study.

Our findings provide neuroimaging evidence that rTMS can modulate the insula despite its relatively deep-seated location. Of course, we cannot exclude the indirect neural activation and/or deactivation of the insula by rTMS given the dense connectivity of the insula with numerous cerebral regions, including the overlying opercula. For instance, intermittent theta-burst stimulation over frontal regions has been shown to indirectly suppress insula activation by modulating fronto-insular connectivity [51]. Addicott et al. [52] also reported an increase in resting-state functional connectivity between the right postcentral gyrus (PCG) and the left insula following both 1 Hz and 10 Hz rTMS over the right PCG. However, the fact that dysgeusia was reported by two participants suggests that the insula was directly stimulated [53]. The symptom of dysgeusia is consistent with previous electrocortical stimulation studies that have reported gustatory hallucinations, such as metallic taste, following middle insula stimulation [54,55,56]. The two participants who experienced gustatory hallucinations showed an increased BOLD signal in the middle insula (see Table 2). This finding supports the role of this insular subregion in the integration of taste-related sensory inputs [53,57,58,59]. The concordance between increased BOLD activity in the mid-insula and the occurrence of gustatory sensations suggests that the activation threshold of this region may be modulated by rTMS, potentially leading to transient changes in sensory perception, as observed in our study.

In our study, both BOLD signal activations and/or deactivations were observed among our participants. This may possibly be explained by differences in the targeted insular subregions. Furthermore, while low-frequency rTMS is widely recognized for its deactivation/inhibitory effects on neural excitation, the mechanism of action of high-frequency stimulation (i.e., ≥5 Hz) remains debated [40]. Indeed, this paradoxical inhibition may result from the activation of inhibitory interneurons, particularly fast-spiking GABAergic circuits, as well as homeostatic plasticity mechanisms that regulate cortical excitability. In addition, the stimulation-induced modulation of functional connectivity may lead to downstream inhibitory effects in interconnected neural networks. Furthermore, these activations and/or deactivations in neural responses appear to be influenced by the length and intensity of the stimulation train, with longer and more intense trains potentially enhancing inhibitory effects through sustained interneuron activation and synaptic plasticity [60,61]. In addition, insular response could differ between individuals depending on baseline excitability [62]. Ko and colleagues [63] suggested that the inconsistent findings in the literature of activation or deactivation across studies may be due to the study populations (i.e., healthy vs. clinical), which may reflect differences in neurochemistry, structural integrity, and connectivity. Finally, it may be possible that 5 Hz rTMS falls into a “grey zone” between excitatory and inhibitory effects. Studies suggest that rTMS responses are frequency-dependent, and certain frequencies, such as 5 Hz, may produce both excitatory and inhibitory results depending on the context, stimulation parameters, and neural circuits [61,64].

The fact that rTMS over the right insula resulted in the modulation of BOLD activity in both hemispheres is not necessarily surprising, considering the connectivity between both insulae and the widespread connectivity of the insula to surrounding lobes [9,65]. In our study, the activated areas are known to be structurally and functionally connected to the insula (see Appendix A, Table A1). It is well established that rTMS has widespread effects beyond the target region. Previous neuroimaging studies have reported that rTMS induces cortical activation in both the stimulated and non-stimulated hemispheres [66,67,68]. Negative BOLD responses in the contralateral hemisphere have been observed after both high-frequency [66] and low-frequency rTMS [69]. Future research should explore the neurotransmitter systems underlying these effects. In addition, it would be valuable to investigate the variability of these responses across different rTMS frequencies and intensities, as well as in different populations.

Choosing the optimal coil is critical for effective deep structure stimulation. A study conducted by Lu and Ueno [70] demonstrated that double-cone, H-coil, and HCA coils show significantly deeper field penetration compared to the conventional “figure of eight” (Fo8) coil, albeit at the expense of inducing higher and more widespread magnetic fields in superficial cortical regions. A double-cone and an HCA coil show a superior ability to stimulate deep brain regions compared to an H-coil. At a depth of 40–60 mm, the volume of brain stimulated above threshold by the H-coil is greater than that stimulated by the double-cone coil, suggesting that the stimulation focus of the double-cone coil is superior at this depth. Conversely, at a depth of 60–80 mm, the volume of brain stimulated above threshold by the H-coil decreases rapidly, while that stimulated by the double-cone coil increases. Currently, there is no consensus on the optimal coil for targeting the insular cortex, as some studies have used a double-cone coil (e.g., D-B80 butterfly coil) [44], an H-coil [39,40,41,42,46,71,72], or a Fo8 coil [52,73,74]. The stimulation intensity varied widely across these studies. Spagnolo et al. [39] reported that a superficial cortical intensity of 145% of MT was required to reach a depth of 4 cm below the scalp. However, such high intensity exceeds safety guidelines for insular stimulation [48,49,50]. Although some studies suggest that higher stimulation thresholds yield better results [40,74], we were unable to apply higher intensities due to the moderate pain induced by pulses at the temple in three participants. We had no choice but to use an MRI-compatible coil for the purpose of these experiments, and thus the intensity of the magnetic field was reduced compared to standard coils due to the thicker casing [75,76].

Several limitations need to be taken into consideration. First, the pulses induced by the coil caused a slight movement of the head during stimulation, making it impossible to obtain clear fMRI images simultaneously with rTMS. Consequently, our results are based on fMRI data acquired immediately post- and between trains of stimulation. High-frequency rTMS has been shown to modulate neuronal activity for up to one hour after stimulation. However, it remains unclear whether this post-stimulation activity mirrors the immediate effects observed during stimulation [50]. Second, we performed only a single high-frequency rTMS session and did not include low-frequency stimulation or a sham condition. Thus, we report the acute effects of rTMS over the insular cortex but cannot infer long-term impacts. Third, the simultaneous rTMS-fMRI recording had inherent challenges, including noise, limited coil positioning on the scalp, restricted movement, and limitations in the choice of coil and rTMS parameters. More specifically, the use of an MRI-compatible TMS coil with a reduced maximal output compared to conventional coils may have limited the depth and intensity of the stimulation. Although stimulation intensity was individualized and neuronavigation was used to optimize coil placement over accessible operculo-insular subregions, the reduced field strength could have attenuated neuromodulatory effects for some participants. Future studies may benefit from using higher-output MRI-compatible coils or advanced coil designs to improve the focal intensity and penetration depth of stimulation in simultaneous rTMS-fMRI settings. Despite the small sample size, our post hoc analysis revealed a substantial average effect size (Cohen’s d = 1.26), indicating significant changes in the BOLD signal in the insular cortex. However, the estimated post hoc statistical power was moderate (57%), reflecting the limited sensitivity typically associated with small-N designs. While these findings support the robustness of the observed effect, they also highlight limitations in statistical generalizability. Replication in larger samples is essential to confirm these results and assess their broader applicability. Even with these limitations, we are confident that our target was effectively stimulated, as evidenced by fMRI images and dysgeusia symptoms specifically associated with the insular cortex.

## 5. Conclusions

In conclusion, this preliminary work demonstrates that 5 Hz high-frequency rTMS can be applied to the insular cortex without significant adverse effects, and that it is possible to reach the insula with TMS using an MRI-B91 coil guided by frameless stereotactic neuronavigation. Concurrent rTMS-fMRI revealed distinct patterns of BOLD signal modulation across insular subregions. Specifically, we observed significant increases in neural activity, reflected by positive BOLD responses, in the left middle insula, bilateral mid-to-inferior insular areas, and the left posterior insula. In contrast, significant decreases in activity, marked by negative BOLD signals, were found in the left ventral anterior insula as well as in bilateral posterior insula regions. Despite the methodological limitations and the small sample size, this study contributes to the growing body of literature on the effects of neuromodulation with rTMS. Further research needs to validate and extend these preliminary observations, including a larger sample size and objective assessments of changes in somatosensory, emotional, and cognitive functions.

## Figures and Tables

**Table 1:** Descriptive characteristics of participants, motor threshold parameters, and excluded subjects.

Subjects	Sex	Motor Threshold Parameters			Reason of Exclusion
		Stimulation Threshold	Stimulation Intensity	Stimulation Amplitude	
SBJ1	F	69	75%	52	-
SBJ2	F	69	75%	52	-
SBJ3	M	77	60%	50	-
SBJ4	F	63	70%	45	-
SBJ5	M	60	60%	36	Stimulation interrupted: Pain at right temple
SBJ6	M	55	70%	39	-
SBJ7	F	-	-	-	Participation declined
SBJ8	M	-	-	-	Exclusion: Contraindication for rTMS-MRI
SBJ9	F	69	75%	52	-
SBJ10	M	-	-	-	Technical issue: rTMS coil malfunction

F = female; M = male. Participants included (*n* = 6) and excluded (*n* = 4).

**Table 2:** BOLD responses in the insular cortex.

	Brainsight Parameters		MNI Coordinates			BOLD Signal Activity	Side	*T*-Value	Cluster Size	FDR- Correction	Sensory Responses Induced by rTMS
	Targeted Insular Subregions	Significant BOLD Changes in the Insular Subregions	x	y	z						
SBJ1	mid-pI	middle/aI	−34	11	−3	A	L	3.17	23	<0.001	Metallic taste
		middle	−41	−1	1	A	L	3.35	10	<0.001	
SBJ2	ventral aI	ventral aI	−34	12	−15	D	L	3.27	67	<0.001	-
SBJ3	aI	middle	−39	−1	−2	A	L	3.32	34	<0.001	-
		mid-inferior	39	−12	−3	A	R	2.73	47	<0.001	
SBJ4	Inferior pI	mid-inferior	−35	−4	−7	A	L	2.36	20	0.05 *	Metallic taste
		pI	39	−25	4	D	R	2.18	131	<0.01	
		pI	−36	−18	6	D	L	1.88	134	<0.01	
		aI	−34	11	−14	D	L	2.56	189	<0.01	
SBJ6	Superior pI	pI	−43	−25	16	A	L	3.22	54	<0.001	-
SBJ9	Inferior pI	Excluded from analysis due to excessive noise contamination									

Clusters ≥ 10 voxels, FDR-corrected at q < 0.01/q < 0.001/q = 0.05 * (trend toward significance). pI: posterior insula; aI: anterior insula; L: left; R: right; A: activation; D: deactivation. Participant # SBJ9 excluded from analysis.

## Data Availability

Dataset available on request from the authors. The data are not publicly available due to ethical reasons.

## Appendix A

**Table A1:** Regions showing significant changes in BOLD signal after rTMS trains over the insula.

Subjects	Side	Anatomic Regions	MNI Coordinates			T-Score	Cluster Size (# of Voxel)
			x	y	z		
SBJ1	L	Inferior frontal gyrus	−30	28	−7	3.84	170
	L	Middle frontal gyrus	−38	40	−9	4.89	154
	L	BA 47	−33	31	−6	3.28	53
	L	Insula	−34	11	−3	3.17	23
	L	Putamen	−23	−3	−1	3.93	22
	L	Lateral globus pallidus	−19	−3	−6	3.52	13
	L	Insula	−41	−1	1	3.35	10
SBJ2	L	Insula/BA 13	−34	13	−9	3.27	67
	L	Temporal pole	−46	8	−8	3.32	50
	L	Superior temporal gyrus	−43	13	−9	3.32	45
SBJ3	L	Midbrain	−1	−31	−15	4.44	340
	L	Insula (middle)	−39	−1	−2	3.32	34
	R	Insula (middle-inferior)	39	−12	−3	2.73	47
	L	Parahippocampal gyrus	−15	−40	−5	3.06	212
	R	Putamen	27	−17	1	2.72	56
	L	Putamen	−28	−15	−2	2.76	57
	R	Globus pallidus	22	−17	1	2.7	67
	R	Supramarginal gyrus	37	−48	29	3.31	33
	R	Post-central gyrus	53	−17	28	2.92	26
SBJ4	L	Superior temporal gyrus *	−47	−11	−1	3.47	27
	L	Insula (middle-inferior) *	−35	−4	−7	2.36	20
	R	Inferior frontal gyrus	−43	20	−15	2.90	534
	R	BA 41	47	−36	13	2.95	86
	L	Superior temporal gyrus	−38	−40	5	3.02	358
	R	Insula (pI)	39	−25	4	2.18	131
	L	Insula (pI)	−36	−18	6	1.88	134
	L	Insula (aI)	−34	11	−14	2.56	189
	R	Globus pallidus	25	−12	1	1.91	81
SBJ6	L	Insula (pI)	−43	−25	16	3.22	54
	R	Posterior cingulate	5	−51	23	4.57	173
	L	Superior temporal gyrus	−37	−45	15	2.81	24

Clusters ≥ 10 voxels, FDR-corrected at q < 0.01/q < 0.001/q = 0.05 * (trend toward significance). pI: posterior insula; aI: anterior insula; L: left; R: right.
